# Factors associated with sexual and reproductive health service utilisation in high migration communities in six Southern African countries

**DOI:** 10.1186/s12889-022-13308-4

**Published:** 2022-05-02

**Authors:** Christine Chawhanda, Jonathan Levin, Latifat Ibisomi

**Affiliations:** 1 School of Public Health, University of Witwatersrand, Johannesburg, South Africa; 2 Nigerian Institute of Medical Research, Lagos, Nigeria

**Keywords:** Migration, Reproductive health, Migrant health, Contraception, Violence, Sexual health

## Abstract

**Background:**

Migration among women has significant health consequences on their access to and utilisation of health services, particularly sexual and reproductive health services. Despite the large quantity of research on migrant health, there is a paucity of research on the factors associated with utilization of modern methods of contraception, intimate partner violence services and sexual and reproductive health (SRH) referral services among non-migrants, internal and international migrant women. Consequently, understanding the factors associated with utilisation of SRH services among women in Southern Africa motivates this study.

**Methods:**

The study uses secondary data from a cross sectional survey conducted in 2018. Logistic regression models were fitted to investigate the factors associated with utilisation of sexual and reproductive health services among 2070 women aged 15–49 years in high migrant communities in six Southern African countries.

**Results:**

Factors found to be associated with current non-use of modern contraceptive methods were country, employment status, educational level, comprehensive knowledge about SRH, comprehensive knowledge about HIV, desire for another child, partner’s age and partner’s educational level. Regarding utilisation of SRH services, important factors were ever denied access to a public healthcare facility, country, marital status and comprehensive knowledge about HIV. Factors associated with utilising IPV services were migration status, age and attitude towards wife beating.

**Conclusion:**

The findings highlight that migration status is associated with utilisation of IPV services. Comprehensive knowledge about SRH and partner characteristic variables were associated with current non-use of modern contraceptive methods. There is a need for SRH programs that can disseminate accurate information about SRH and encourage male involvement in SRH related issues. In addition, the SRH programs should target all women regardless of their migration status, age, educational level and marital status.

**Supplementary Information:**

The online version contains supplementary material available at 10.1186/s12889-022-13308-4.

## Background

Globally, internal migration was estimated at 740 million in 2009 and increased to 763 million in 2015 [1–3]. Similarly, an increase in international migration has been documented. International migration worldwide was estimated to be 214 million in 2010, rose to approximately 244 million in 2015 and to about 258 million in 2017 [2, 4–6]. It is estimated that one billion people are residing outside of their original place of birth or residence [7]. In Africa, there were approximately 25 million international migrants in 2017, an increase of 3% since the year 2000 [5]. Within Africa, the share of international women migration increased from 45% in 2010 to 48% in 2017 [2, 5]. Most of the migrants are in the reproductive age group (15—49 years) with the average age of international migrants being 39 years [2]. The migration of people of the reproductive age group has important implications for public health and in particular, access to and utilisation of SRH services.

Existing literature has shown that inequalities exist in utilisation of SRH services between migrants and non-migrants. Being a migrant has been associated with compromised utilisation of health care, including SRH services [7–12]. Literature has documented low contraceptive use, high experience of intimate partner violence (IPV), high maternal morbidity and mortality, high abortion rates and abortion complications and high HIV prevalence among migrant women compared to non-migrant women [8–10, 13–24].

Several barriers to utilisation of SRH services by migrant women have been documented. These include religious and cultural beliefs, financial constraints, fear of deportation among migrants who do not possess legal documents, language barriers, lack of SRH information, and the attitude of service providers [4, 8, 12, 14, 15, 21, 23–30]. Further, lower educational level, being unmarried, financial instability and high costs of services for migrants are factors associated with lower utilisation of contraception methods and HIV services [21, 23, 31, 32]. The aforementioned findings were mainly collated from studies that focused on qualitative exploration of attitudes, perceptions and experiences of migrants in accessing SRH services [12, 15, 25, 33–35]. In addition, in Southern Africa, research has been conducted on migrant commercial farm workers focusing on determinants of condom use in South Africa and unmet need for contraception among internal female migrants in Zambia, [31, 36]. Similarly, a few mixed methods studies were conducted which focused on access to HIV care and treatment among migrants in Lesotho and South Africa [37].

There is a dearth of information on migrant women’s access to and utilisation of IPV services and SRH referrals. In addition, emphasis has been on international migrants, neglecting the needs of internal migrants although they may also face challenges in utilisation of SRH services. From previous studies, little is known concerning the influence of partner characteristics, SRH decision making power, intimate partner violence (IPV), comprehensive knowledge about HIV and comprehensive knowledge about SRH on migrant women’s access to SRH services as they influence health seeking behaviour of women. To broaden the understanding of utilization of SRH services by migrants it is vital to contextually explore the factors migrants face in the communities they reside and work in. Against this backdrop, the objective of this study is to estimate the rates of utilisation and factors associated with the utilisation of SRH services among women in high migrant communities in the Kingdom of Eswatini, Lesotho, Malawi, Mozambique, South Africa and Zambia. As such, it is hypothesised that migrant women, women with lower comprehensive knowledge about SRH, lower comprehensive knowledge about HIV and who have younger partners are less likely to utilise SRH services. The hypothesis is premised on that migrant women face challenges in utilising SRH services in the host country and women who lack SRH and HIV knowledge rarely utilise SRH and HIV services. The focus is on utilization of the following SRH services—modern contraceptive methods, IPV services and SRH referral services.

**Fig. 1:**
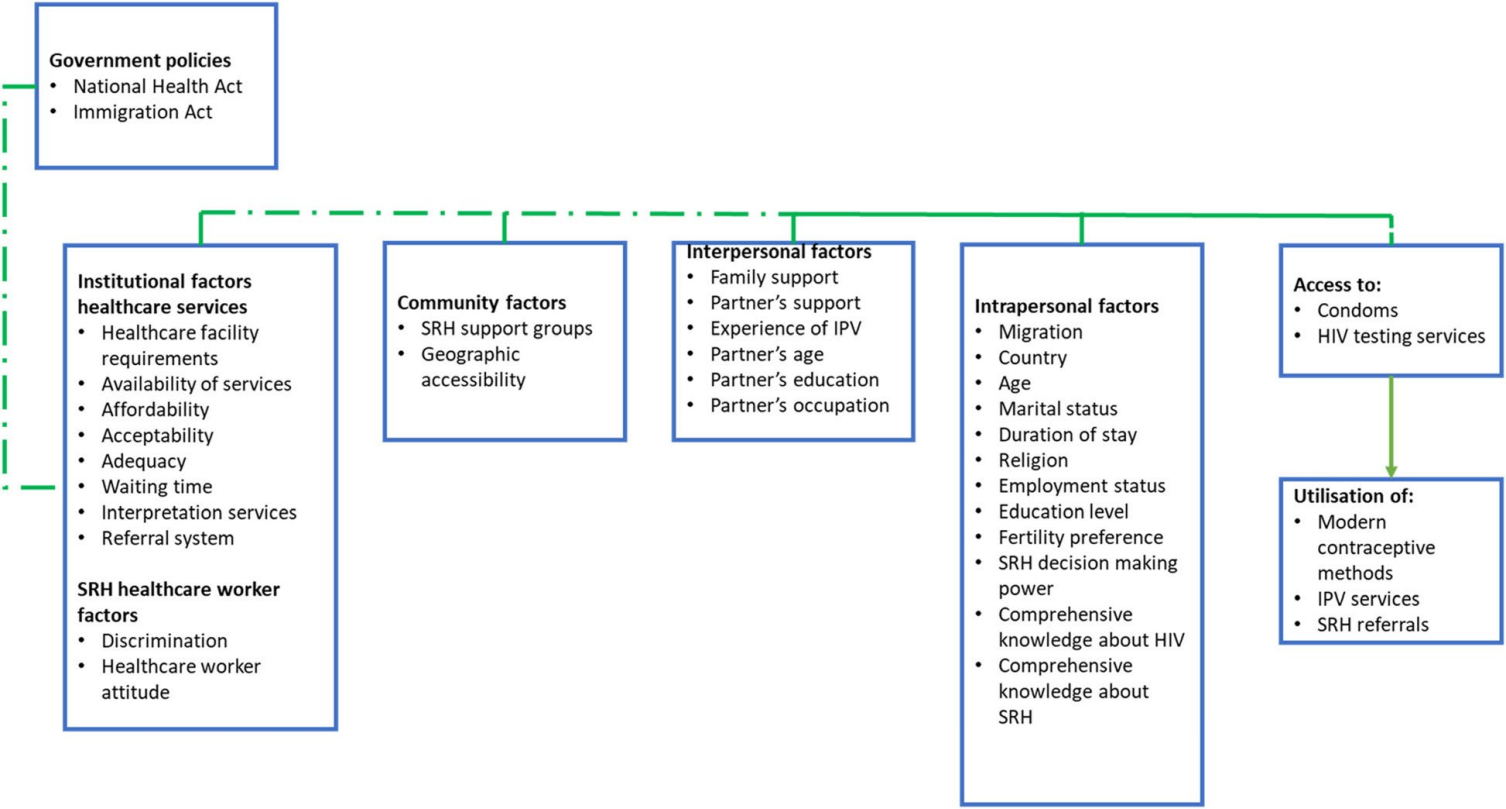
The conceptual framework

Figure 1 shows the conceptual framework guiding this study. The conceptual framework is drawn from The Social Ecological Theory of McLeroy et al. (1988) and the Health Access Framework of Obrist et al. (2007). It shows that factors at different levels influence women’s decisions to access and utilise SRH services and that access to SRH is a prerequisite for women’s utilisation of SRH services. This paper is from the first author’s PhD work, hence the paper focuses on some of the interpersonal factors and intrapersonal factors while other sections are investigated and explored in other papers. The conceptual framework shows different levels, however the study does not use multilevel modelling due to an error in data collection as village identifiers were not collected.

## Data and methods

This study is a secondary analysis of the data from a baseline survey conducted as part of the SRHR-HIV Knows no Borders Project, a collaboration between the International Organization for Migration (IOM), Save the Children Netherlands (SCN) and University of the Witwatersrand’s School of Public Health (WSPH) (consortium partners). The survey was conducted in 2018 in 10 high migration communities in six Southern African countries, namely Hhohho (The Kingdom of Eswatini), Maputsoe (Lesotho), Mwanza and Mchinji (Malawi), Chifunde and Ressano Garcia (Mozambique), Ekurhuleni and Nkomazi (South Africa), Chipata and Katete (Zambia). The rationale for selecting the countries and the communities in particular by the SRHR-HIV Project is that they are labour and transport corridors for migration and have major inflows of migrants both from within the countries themselves and from other African countries. The SRHR-HIV knows no Borders Project consortium obtained ethics approval from the relevant authorities in each of the six countries while permission to use the data for this study was obtained from the SRHR-HIV Knows no Borders Project consortium. To conduct this secondary data analysis, formal approval was obtained from Human Research Ethics Committee-Medical of the University of Witwatersrand (Clearance Certificate Number M190601). The role of the first author in the baseline survey was to train the data capturers and supervise the data collection process while the second and third authors were part of the conceptualization of the project, design of study tools and academic supervisor of the first author.

The target population for the baseline survey were adolescent and young people (AYPs), sex workers (SWs), migrants, truck drivers and the settled population. Eligible participants for the baseline survey included women of reproductive age (15–49 years) and men aged 15–59 years who were regular household members, visitors were excluded from the study. A sample size of 300 per study site was used of whom approximately two-thirds were female, which yielded a pooled sample size of 2070 females for the 10 sites in six countries. A total sample size of 2070 would be sufficient to detect an absolute difference of 12% in the rate of utilisation of a given SRHR service (e.g. use of a modern contraceptive method) between two groups, e.g. international migrants and non-migrants, as being statistically significant at the 5% level with 80% power, based on the following assumptions a) roughly equal numbers of women are in each of the three migration status categories and b) the effect of the clustering of women within sites results in a moderate Design Effect (DEFF) of 1.5.

To facilitate access to the SWs and the mobile populations, a rapid mapping exercise was carried out at each site to identify hotspots for SWs, truck stops and sleeping places of mobile populations. Within each selected project site, random selection of clusters (villages/wards) and listing of buildings and households was performed in each cluster using a household schedule form. To avoid errors in uniquely numbering each building/household, listing of the buildings and households was done sequentially. In a household with more than 3 eligible participants, a maximum of three respondents were randomly selected. Snowball sampling was used to recruit sex workers. In this study, data for 2070 women aged 15–49 years that participated in the SRHR-HIV project baseline survey is used. The data from eligible women was collected using the individual women questionnaire which collected information on socio-demographic information, marital status, sexual activity, family planning/contraception, pregnancy and reproduction, fertility preferences, HIV/AIDS and STIs, IPV, gender roles and other health related issues including referrals.

### Measurement of variables

#### Outcome variables

There are three outcome variables that measure utilisation of SRH services, namely current non-use of modern contraceptive methods, utilisation of IPV services, and utilisation of SRH referral services. Current non-use of modern contraceptive methods is defined as currently not using any modern method to delay or avoid getting pregnant. The reason for analysing non-use of modern contraceptive methods is due to the difficulty in interpreting ORs when the prevalence of the outcome is high. Utilisation of IPV services is defined as having used or sought any formal services related to physical or sexual violence perpetrated by their intimate partners. The variable was derived from the question “Have you ever told anyone about any of the abuse or sought help? (Yes/No)” and those who responded Yes were further asked from who/where? Women were regarded as having sought help if they sought help from medical personnel, police, lawyer, social worker, NGOs marriage counsellors, the victim support unit or community leaders and not having sought help if they sought help from their own family, partner’s family, neighbours or friends. The question was asked to women who reported experiencing physical or sexual violence in the past 12 months. Utilisation of SRH referral services was derived from the question as to which SRH-HIV services the woman had been referred for since the services were not offered at a health facility that she had visited (Facilities in community, facilities outside community). Respondents who responded to have been referred either to facilities in or outside the community were further asked if they went for the referred services.

#### Explanatory variables

The main explanatory variable was migration status, categorised as non-migrants, internal migrants or international migrants. Comprehensive knowledge about SRH is a discrete quantitative variable taking integer values between 0 and 2 inclusive, and is derived from questions about effective ways of preventing pregnancy, rejecting two or more misconceptions about pregnancy and knowing fertile days. A correct answer on each question is scored 1 and incorrect answer/don’t know is scored 0. Comprehensive knowledge about HIV is a discrete quantitative variable taking integer values between 0 and 3 inclusive, which is defined as respondents having knowledge about HIV transmission, rejecting two or more HIV myths and knowing that a healthy looking person can be HIV positive. It is derived from a set of questions and a correct answer on each question is scored 1 and incorrect answer/don’t know is scored 0.

SRH decision making power is a discrete quantitative variable which takes integer values between 0 and 5 and is derived from the responses to five questions which measure a woman’s ability to decide about timing of sexual intercourse, ability to refuse sex when not in the mood, timing of pregnancy and mode of child delivery. Women who responded that they have the power to decide for all five questions were given a score of five while those who reported that they did not have the power to decide for each of the five questions were given a score of zero.

Experience of IPV refers to encountering physical or sexual violence from an intimate partner in the past 12 months. It is derived from the following questions: did your (last) intimate partner ever do any of the following things to you in the last 12 months: for physical violence- push you, shake you or throw something at you, slap you, twist your arm or pull your hair, punch you with his fist or something that could hurt you, kick, drag you or beat you up, try to choke you or burn you on purpose and threaten or attack you with a knife. For sexual violence, physically force you to have sexual intercourse with him when you did not want to, and physically force you to perform any other sexual acts you did not want to. A respondent was regarded as experiencing IPV if they experienced any of the instances of physical or sexual violence and as not experiencing IPV if they did not experience any physical violence nor any sexual violence.

The explanatory variables for current non-utilisation of modern contraceptive methods are: age, marital status, educational level, employment status, country, duration of stay in the community, religion, fertility preference, comprehensive knowledge about SRH, comprehensive knowledge about HIV, SRH decision making power, partner’s age, partner’s educational level, partner’s occupation and experience of IPV.

The explanatory variables for utilisation of SRH referral services are: age, marital status, educational level, employment status, ever denied access to a public healthcare facility, country, duration of stay in the study site, religion, comprehensive knowledge about SRH, comprehensive knowledge about HIV, SRH decision making power, partner’s age, partner’s educational level, and partner’s occupation.

The explanatory variables for utilisation of IPV services are: age, marital status, educational level, employment status, country, duration of stay, religion, witnessing parental violence, attitude towards wife beating, comprehensive knowledge about SRH, comprehensive knowledge about HIV, SRH decision making power, partner’s age, partner’s educational level and partner’s occupation.

### Data analysis

Descriptive statistics were used to summarize the utilization of SRH services by the explanatory factors. Three multivariable logistic regression models were fitted to investigate the factors that were independent predictors of the utilization of SRH services. Additional analyses were carried out restricted to women who were partnered. Migration status is the primary exposure variable and it was included in all the models and other variables that were found to be significant in the univariable model at *P* = 0.20 and the variables that were found to be relevant in literature were used in the multivariable model. For the sub-analysis, in addition to partner characteristic variables that were found to be relevant in the literature, all the explanatory variables that were used in the main analysis were included. All data management and data analysis was carried out using Stata release 15.0

## Results

Table 1 shows the distribution of selected characteristics of the respondents broken down by whether or not they used modern contraceptive methods. Regarding age, women who used modern contraceptive methods were slightly younger on average than women who did not use modern contraceptive methods (*P* = 0.05). Educational level differed between women who used modern contraceptive methods and women who did not; a higher proportion of women who used modern contraceptive methods had secondary or higher education than among women who did not use modern contraceptive methods (*P* < 0.001). Similarly, employment status differed between women who used modern contraceptive methods and women who did not. Women who used modern contraceptive methods were more likely to be employed than women who did not use modern contraceptive methods (*P* < 0.01). Comprehensive knowledge about SRH differed between women who used modern contraceptive methods and women who did not. Women who used modern contraceptive methods had higher comprehensive knowledge about SRH on average than women who did not use modern contraceptive methods (*P* < 0.001). Similarly, comprehensive knowledge about HIV differed between women who used modern contraceptive methods and women who did not. On average, women who used modern contraceptive methods had higher comprehensive knowledge about HIV than women who did not use modern contraceptive methods (*P* < 0.001).

**Table 1:** Distribution of utilisation of modern contraceptive methods by respondents’ selected characteristics in high migration communities of six Southern African countries *N* = 1677

Variable	Level	Utilisation of modern contraceptive methods			*P*-value
		**Total**	**utilised**	**Not utilised**	
		***N* = 1677 (%)**	***n* = 1023(%)**	***n* = 654(%)**	
**Migration status**	Non-migrant	1129 (67.3)	680 (66.5)	449(68.6)	0.15
	Internal migrants	333(19.9)	218 (21.3)	115(17.6)	
	International migrants	215(12.8)	125(12.2)	90(13.8)	
**Duration of stay in community**	Mean duration (SD)	20.1(12.8)	19.6(12.5)	20.8 (13.2)	*P* < 0.001
**Country**	Lesotho	209(12.5)	115(11.2)	94(14.4)	0.02
	Malawi	411(24.5)	280(27.3)	131(20.0)	
	Mozambique	304(18.1)	179(17.5)	125(19.1)	
	South Africa	265(15.8)	157(15.4)	108(16.5)	
	Eswatini	156(9.3)	94(9.2)	62(9.5)	
	Zambia	332(19.8)	198(19.4)	134(20.5)	
**Age**	Mean (SD)	26.9(8.3)	26.6(7.7)	27.4(9.3)	0.05
**Marital status**	Never married	716(42.0)	441(43.1)	275(42.1)	0.31
	Married	666(38.4)	415(40.6)	251(38.4)	
	Formerly married	292(19.1)	167(16.3)	125(19.1)	
	Missing	3(0.5)	0(0.0)	3(0.5)	
**Educational level**	Primary/lower	686(40.9)	399(39.0)	287(43.9)	*P* < 0.001
	Secondary/higher	982(58.6)	620(60.6)	362(55.4)	
	Missing	9(0.5)	4(0.4)	5(0.7)	
**Employment status**	Unemployed	804 (48.0)	465(45.5)	339(51.8)	*P* < 0.01
	Employed	872(52.0)	558(55.5)	314(48.0)	
	Missing	1(0.0)	0(0.0)	1(0.2)	
**Religion**	Catholic	556(33.1)	321(31.4)	235(35.9)	0.09
	Other Christians	957(57.1)	605(59.1)	352(53.8)	
	Other religion	36(2.2)	97(9.5)	67(10.2)	
**Desire for another child**	Have a child < 2 years	75(4.5)	51(5.0)	24(3.7)	*P* < 0.001
	Have a child > = 2 years	261(15.6)	181(17.7)	80(12.2)	
	No more	607(36.2)	388(37.9)	219(33.5)	
	Unsure	37(2.2)	23(2.3)	14(2.1)	
	Don’t have a partner	137(8.2)	76(7.4)	61(9.3)	
	Missing	560(33.4)	304(29.7)	256(39.1)	
**Comprehensive knowledge about SRH**	Mean (SD)	0.90(0.45)	0.93(0.43)	0.84(0.46)	*P* < 0.001
**Comprehensive knowledge about HIV**	Mean (SD)	2.32(0.88)	2.39(0.81)	2.21(0.97)	*P* < 0.001

Table 2 shows the distribution of selected characteristics of the respondents broken down by whether or not they have utilised SRH referral services. It is shown that of the 796 women who reported being referred for SRH services, 363 utilised the referral services while 433 did not. Migration status differed marginally between women who utilised SRH referral services and women who did not. Compared to women who did not utilize referrals, a higher proportion of women who utilized referrals were international migrants and a lower proportion were non-migrants. Ever denied access to a public healthcare facility differed between women who utilised SRH referral services and women who did not. Among women who utilised SRH referral services 62% had access to a healthcare facility, while among those who did not utilise SRH referral services 76% had access (*P* < 0.001).

**Table 2:** Distribution of utilisation of SRH referral services by respondents’ selected characteristics in high migration communities of six Southern African countries *N* = 796

Variable	Level	Utilisation of SRH referral services			*P*-value
		**Total**	**Utilised**	**Not utilised**	
		***N* = 796**	***n* = 363(%)**	***n* = 433 (%)**	
**Migration status**	Non-migrant	539(67.7)	231(63.6)	308(71.1)	0.07
	Internal migrants	109(13.7)	54(14.9)	55(12.7)	
	International migrants	148(18.6)	78(21.5)	70(16.2)	
**Duration of stay in community**	Mean duration of stay (SD)	19.8(12.4)	19.7(12.8)	19.9(12.1)	0.81
**Ever denied access to a public healthcare facility**	Yes	239(30.0)	135(37.2)	104(24.0)	*P* < 0.001
	No	557(70.0)	228(62.8)	329(76.0)	
**Country**	Lesotho	7(0.89)	0(0.0)	7(1.6)	*P* < 0.001
	Malawi	198(24.9)	82(22.6)	116(26.8)	
	Mozambique	176(22.1)	60(16.5)	116(26.8)	
	South Africa	130(16.3)	75(20.7)	55(12.7)	
	Eswatini	156(19.6)	81(22.3)	75(17.3)	
	Zambia	129(16.2)	65(17.9)	64(14.8)	
**Age**	Mean age (SD)	25.9(19;30)	27(8.2)	24.9(8.4)	*P* < 0.001
**Marital status**	Never married	409(51.5)	167(46.0)	242(55.9)	0.02
	Married	274(34.5)	136(37.5)	138(31.9)	
	Formerly married	112(14.0)	60(16.5)	52(12.0)	
	Missing	1(0.0)	0(0.0)	1(0.2)	
**Educational level**	Primary/lower	338(42.5)	143(39.5)	195(45.0)	0.12
	Secondary/higher	457(57.5)	219(60.5)	238(55.0)	
**Employment status**	Unemployed	360(45.3)	159(43.8)	201(46.4)	0.71
	Employed	435(54.7)	204(56.2)	231(53.4)	
	Missing	1(0.0)	0(0.0)	1(0.2)	
**Religion**	Catholics	243(30.5)	102(28.1)	141(32.6)	0.31
	Other Christians	472(59.3)	220(60.6)	252(58.2)	
	Other religion	81(10.2)	41(11.3)	40(9.2)	
**Comprehensive knowledge about SRH**	Mean ( SD)	0.85(0.47)	0.88(0.48)	0.82(0.46)	0.06
**Comprehensive knowledge about HIV**	Mean(SD)	2.29(0.93)	2.39(0.85)	2.21(0.99)	*P* < 0.01

Regarding age, on average, women who utilised SRH referral services were older than women who did not utilise SRH referral services (*P* < 0.001). Religion differed between women who utilised SRH referral services and women who did not. Women who utilized SRH referrals were more likely to be “other Christian” while those who did not were more likely to be Catholic or Other religion (*P* < 0.01). Comprehensive knowledge about SRH differed between women who utilised SRH referral services and women who did not. Women who utilised SRH referral services had higher comprehensive knowledge about SRH on average than women who did not (*P* = 0.03). Similarly, comprehensive knowledge about HIV differed between women who utilised SRH referral services and women who did not. Women who utilised SRH referral services had higher comprehensive knowledge about HIV on average than women who did not utilise SRH referral services (*P* < 0.01).

Table 3 shows the distribution of selected characteristics of the respondents broken down by whether or not they have utilised IPV services. It is shown that, of the 525 women who experienced IPV, 204 utilised IPV services while 321 did not. Duration of stay in the study site differed between women who utilised IPV services and women who did not. Women who utilised IPV services had stayed longer in the site (community) on average than women who did not utilise IPV services (*P* = 0.03). Similarly, age differed between women who utilised IPV services and women who did not. Women who utilised IPV services were older on average than those who did not utilise IPV services (*P* < 0.001). Employment status differed between women who utilised IPV services and women who did not, with women who utilised IPV services more likely to be employed than women who did not utilise IPV services (*P* < 0.001). Comprehensive knowledge about HIV differed between women who utilised IPV services and women who did not, with women who utilised IPV services having higher comprehensive knowledge about HIV on average than women who did not utilise IPV services (*P* < 0.01).

**Table 3:** Distribution of utilisation of IPV services by respondents’ selected characteristics in high migration communities of six Southern African countries *N* = 525

Variable	Level	Utilisation of IPV services			*P*-value
		**Total**	**Utilised**	**Not utilised**	
		***N* = 525**	***n* = 204 (%)**	***n* = 321 (%)**	
**Migration status**	Non-migrant	361(68.8)	144(70.6)	217(67.6)	0.56
	Internal migrants	100(19.0)	39(19.1)	61(19.0)	
	International migrants	64(12.2)	21(10.3)	43(13.4)	
**Duration of stay**	Mean duration (SD)	20(12.5)	21.1(13.3)	19.3(11.9)	0.03
**Country**	Lesotho	24(4.6)	11(5.4)	13(4.1)	*P* < 0.01
	Malawi	172(32.8)	76(37.2)	96(29.9)	
	Mozambique	59(11.2)	15(7.4)	44(13.7)	
	South Africa	46(8.8)	18(8.8)	28(8.7)	
	Eswatini	101(19.2)	28(13.7)	73(22.7)	
	Zambia	123(23.4)	56(27.5)	67(20.9)	
**Age**	Mean age (SD)	26.2(7.9)	27.9(8.1)	25.2(7.7)	*P* < 0.001
**Marital status**	Never married	218(41.5)	60(29.4)	158(49.2)	*P* < 0.001
	Married	201(38.3)	87(42.7)	114(35.5)	
	Formerly married	106(20.2)	57(27.9)	49(15.3)	
**Educational level**	No education	48(9.1)	16(7.9)	32(10.0)	0.44
	Primary	190(36.2)	80(39.2)	110(34.3)	
	Secondary/higher	287(54.7)	108(52.9)	179(55.8)	
**Employment status**	Unemployed	274(52.2)	80(39.2)	171(53.3)	*P* < 0.001
	Employed	251(47.8)	124(60.8)	150(46.7)	
**Comprehensive knowledge about SRH**	Mean(SD)	0.91(0.45)	0.92(0.41)	0.90(0.47)	0.76
**Comprehensive knowledge about HIV**	Mean (SD)	2.36(0.84)	2.5(0.71)	2.26(0.91)	*P* < 0.001
**Witnessing parental violence**	No	199(37.9)	76(37.3)	123(38.3)	0.81
	Yes	326(62.1)	128(62.7)	198(61.7)	
**Attitude towards wife beating**	No	249(47.4)	119(58.3)	130(40.5)	*P* < 0.001
	Yes	276(52.6)	85(41.7)	191(59.5)	
**Partner’s age**	Mean age (SD)	30.2(9.4)	31.7(10.5)	29.2(8.4)	0.01
**Partner’s educational level**	No education	122(25.1)	39(19.2)	83(25.9)	*P* < 0.01
	Primary	101(20.8)	51(25.0)	50(15.6)	
	Secondary/higher	263(54.1)	108(52.9)	155(48.3)	
	Missing	39(7.0)	6(2.9)	33(10.3)	
**Partner’s occupation**	None	30(6.3)	11(5.7)	19(6.7)	0.10
	Agriculture	59(12.4)	20(10.4)	39(13.7)	
	Technical/managerial	61(12.8)	17(8.8)	44(15.5)	
	Skilled manual	112(23.5)	47(24.4)	65(22.9)	
	Unskilled manual	215(45.1)	98(50.8)	11(41.2)	
**SRH decision making power**	Mean (SD)	2.4(1.8)	2.9(1.7)	2.1(1.9)	*P* < 0.001

Attitude towards wife beating differed between women who utilised IPV services and women who did not. A higher proportion of women who utilised IPV services resided in communities that do not tolerate wife beating compared to women who did not utilise IPV services (*P* < 0.001). Regarding partner characteristics, partner’s age differed between women who utilised IPV services and women who did not; women who utilised IPV services had older partners on average than women who did not utilise IPV services (*P* < 0.01). Similarly, the influence of partner’s educational level differed between women who utilised IPV services and women who did not. A higher proportion of women who utilised IPV services had partners with secondary or higher education than women who did not utilise IPV services (*P* < 0.01). Sexual and reproductive health decision making power differed between women who utilised IPV services and women who did not. Women who utilised IPV services had more SRH decision making power on average compared to women who did not utilise IPV services (*P* < 0.001). In addition, being afraid of one’s partner differed between women who utilised IPV services and women who did not. A higher proportion of women who utilised IPV services were afraid of their partners compared to women who did not utilise IPV services (*P* < 0.001).

### Utilisation of modern contraceptive methods

Table 4 presents the prevalence and the factors associated with current non-use of modern contraceptive methods by all women. Adjusting for other factors, there was no evidence of an association between migration status and non-utilisation of modern contraceptive methods (*P* = 0.40). Country was significantly associated with current non-use of modern contraceptive methods (*P* = 0.028).

**Table 4:** Factors associated with current non-use of modern contraceptive methods among women in high migration communities of six Southern African countries

Variable	Level	All women			*P*-value
		**Prevalence of modern contraceptive non-use n (%)**	**Unadjusted** **OR (95% CI)**	**Adjusted OR** **OR (95% CI)**	
		**654 (39.0)**		***n* = 1665**	
**Migration status**	Non-migrant	449(39.8)	1.00	1.00	0.40
	Internal migrants	115(34.5)	0.79(0.62–1.03)	0.87(0.55–1.36)	
	International migrants	90(41.9)	1.09(0.81–1.47)	1.13 (0.7–1.83)	
**Duration of stay in community**			1.04(1.00–1.08)	1.03(0.95–1.13)	0.46
**Country**	South Africa	108(40.8)	1.00	1.00	0.028
	Lesotho	94(45.0)	1.19( 0.82–1.71)	1.42(0.93–2.15)	
	Malawi	131(31.9)	0.68 (0.49–0.94)	0.73(0.50–1.07)	
	Mozambique	125(41.1)	1.02(0.73–1.42)	0.85(0.58–1.26)	
	Eswatini	62(39.7)	0.96(0.64–1.44)	1.08(0.70–1.65)	
	Zambia	134(40.4)	0.98(0.71–1.37)	0.87(0.59–1.28)	
**Age**			1.06( 1.01–1.13)	1.10(1.01–1.22)	0.06
**Marital status**	Never married	275(38.4)	1.00	1.00	0.05
	Married	251(37.7)	0.97 (0.78–1.21)	1.20( 0.90–1.58)	
	Formerly married	125 (42.8)	1.20 (0.91–1.58)	1.51( 1.08–2.14)	
**Educational level**	Primary/lower	287(41.8)	1.00	1.00	0.045
	Secondary/higher	362(36.9)	0.81( 0.66–0.99)	0.79(0.63–1.00)	
**Employment status**	Employed	314(36.0)	1.00	1.00	0.006
	Unemployed	339(42.2)	1.30 (1.06–1.58)	1.34(1.09–1.67)	
**Religion**	Other Christians	352(36.8)	1.00	1.00	0.23
	Catholics	235(42.3)	1.26(1.02–1.56)	1.22(1.03–1.53)	
	Other religion	67(40.9)	1.19(0.85–1.66)	1.04(0.72–1.50)	
**Desire for another child**	Have a child < 2 years	24(32.0)	1.00	1.00	*P* < 0.001
	Have a child > 2 years	80(30.7)	0.94(0.54–1.63)	1.03(0.58–1.81)	
	No more	219(36.1)	1.20(0.72–2.00)	0.98(0.58–1.67)	
	Unsure	14(37.8)	1.29(0.57–2.94)	1.33(0.57–3.09)	
	No partner	61(44.5)	1.71(0.94–3.08)	2.19(1.16–4.12)	
	Missing	256(45.7)	1.79(1.07–2.99)	2.07(1.21–3.53)	
**Comprehensive knowledge about SRH**			0.62 (0.50–0.78)	0.73( 0.57–0.93)	0.010
**Comprehensive knowledge about HIV**			0.80(0.72–0.89)	0.87(0.77–0.98)	0.023

Employment status was significantly associated with modern contraceptive non-use after controlling for other factors (*P* = 0.006). Unemployed women had 34% higher odds of not using modern contraceptive methods compared to employed women (aOR = 1.34, 95% CI 1.09–1.67). There was a significant association between desire for another child and current non-use of modern contraceptive methods (*P* < 0.001). Compared to women who prefer to have a child within two years, women with no partners had higher odds of current non-use of modern contraceptive methods (aOR = 2.19; 95% CI 1.16–4.12).

There was a significant association between comprehensive knowledge about SRH and current non-use of modern contraceptive methods (*P* = 0.010). In the multivariable model, with a unit increase in comprehensive knowledge about SRH, the odds of current non-use of modern contraceptive methods decreased by 27% (aOR = 0.73, 5% CI 0.57–0.93). Similarly, comprehensive knowledge about HIV was significantly associated with current non-use of modern contraceptive methods (*P* = 0.023). A unit increase in comprehensive knowledge about HIV decreased the odds of current non-use of modern contraceptive methods by 13% (aOR = 0.87; 95% CI 0.77–0.98). There was a marginal association between age and current non-use of modern contraceptive (*P* = 0.06). The adjusted result shows that with a five year increase in age, the odds of current non-use of modern contraceptive methods increased by 10% (aOR = 1.10, 95% CI 1.01–1.22).

Supplementary Table 1 shows the factors associated with current non-utilisation of modern contraceptive methods in a sub-analysis restricted to women who were currently partnered. Similar to the results in Table 4a, Supplementary table 1 shows no evidence of an association between migration status and current non-use of modern contraceptive methods after adjusting for other factors (*P* = 0.81). Regarding partner’s characteristic variables, partner’s age was significantly associated with current non-use of modern contraceptive methods (*P* = 0.013). Compared to women with partners aged 24 years and below, women with partners aged 25–34 years, 35 years and above and those who did not know the age of their partners had lower odds of current non-use of modern contraceptive methods (aOR = 074; 95% CI 0.49–1.10), (aOR = 0.54, 95% CI 0.33–0.90) and (aOR = 0.66, 95% CI 0.40–0.1.10) respectively. In addition, partner’s education was marginally associated with current non-use of modern contraceptive methods (*P* = 0.05). Compared to women whose partners had primary or lower education, women with partners with secondary/higher education had 28% lower odds of current non-use of modern contraceptive methods (aOR = 0.72, 95% CI 0.50–0.99).

### Utilisation of SRH referral services

Table 5 shows the factors associated with utilisation of SRH referral services by all women. Adjusting for other factors, there was no evidence of an association between migration status and utilisation of SRH services (*P* = 0.95). Ever denied access to healthcare facility was associated with utilisation of SRH referral services (*P* < 0.001). All women who reported to have been denied access to a healthcare facility had higher odds of utilising SRH referral services compared to women who were never denied access to healthcare facility (aOR = 2.49; 95% CI 1.66–3.73). Country was strongly associated with utilisation of SRH referral services (*P* < 0.001). Women in Malawi (aOR = 0.23; 95% CI 0.13–0.42); Mozambique (aOR = 0.38; 95% CI 0.22–0.65) and Eswatini (aOR = 0.60; 95% CI 0.36–0.99) had lower odds of utilising SRH referral services compared to women in South Africa. Lesotho is empty in the analysis because all the participants who were referred did not utilise the referral services resulting in perfect prediction.

**Table 5:** Factors associated with utilisation of SRH referral services among all women in high migration communities of six Southern African countries

**Variable**	**Level**	**Prevalence of utilisation of SRH referral services**	**Unadjusted OR (95% CI)**	**Adjusted OR (95% CI)**	***P*-value**
		***N* = 362(45.5)**		***N* = 787**	
**Migration status**	Non-migrant	230(42.8)	1.00	1.00	0.95
	Internal migrants	54(49.5)	1.31(0.87–1.98)	1.08(0.57–2.07)	
	International migrants	78(52.7)	1.49 (1.03–2.14)	1.11(0.58–2.12)	
**Duration of stay**			0.99(0.94–1.05)	0.93(0.81–1.07)	0.31
**Ever denied access to a public healthcare facility**	No	228(40.9)	1.00	1.00	*P* < 0.001
	Yes	135(56.5)	1.87(1.38–2.54)	2.49(1.66–3.73)	
**Country**	South Africa	75(57.7)	1.00	1.00	*P* < 0.001
	Lesotho	0(0)	Empty	Empty	
	Malawi	82(41.4)	0.52(0.33–0.81)	0.23(0.13–0.42)	
	Mozambique	60(34.1)	0.38(0.24–0.61)	0.38(0.22–0.65)	
	Eswatini	81(51.9)	0.79(0.50–1.27)	0.60(0.36–0.99)	
	Zambia	65(50.0)	0.74(0.46–1.22)	0.58(0.33–1.03)	
**Age**			1.15(1.06–1.26)	1.14(0.98–1.32)	0.08
**Marital status**	Never married	167(40.8)	1.00	1.00	0.006
	Married	135(49.5)	1.43 (1.05–1.94)	1.61 (1.09–2.36)	
	Formerly married	60(53.5)	1.67 (1.10–2.55)	2.14(1.29–3.55)	
**Educational level**	Primary/lower	143(42.3)	1.00	1.00	0.78
	Secondary/higher	219(47.9)	1.25(0.95–1.67)	1.05(0.74–1.48)	
**Employment status**	Unemployed	204(46.9)	1.00	1.00	0.43
	Employed	158(44.0)	0.90(0.68–1.19)	0.88(0.65–1.21)	
**Religion**	Catholic	102(42.0)	1.00	1.00	0.80
	Other Christians	220(46.6)	1.21(0.88–1.65)	1.09(0.77–1.54)	
	Other religion	41(50.6)	1.42(0.86–2.45)	1.19(0.68–2.08)	
**Comprehensive knowledge about SRH**			1.32(0.98–1.78)	1.14(0.81–1.61)	0.44
**Comprehensive knowledge about HIV**			1.24(1.06–1.44)	1.18(0.99–1.42)	0.06

The association between utilisation of SRH referral services and age was marginally significant (*P* = 0.083). A five year increase in age increased the odds of utilising SRH referral services by 14% (aOR = 1.14, 95% CI 0.98–1.32). Marital status was significantly associated with utilisation of SRH referral services (*P* = 0.006). Compared to never married women, married women and formerly married women had higher odds of utilising SRH referral services (aOR = 1.61, 95% CI 1.09–2.36) and (aOR = 2.14, 95% CI 1.29–3.55) respectively. There was a marginal association between comprehensive knowledge about HIV and utilisation of SRH referral services (*P* = 0.06). A unit increase in comprehensive knowledge about HIV increased the odds of utilising SRH referral services by 18% (aOR = 1.18; 95% CI 0.99–1.42).

Supplementary table 2 shows the factors associated with utilisation of SRH referral services in a sub-analysis restricted to women who were currently partnered. Similar to the results in Table 5, the results in supplementary table 2 shows no evidence of an association between migration status and utilisation of SRH services among partnered women (*P* = 0.54). In addition, there was no evidence of an association of partner characteristic variables, SRH decision making power, experience of IPV and utilisation of SRH referral services. However, women whose partners were into technical/managerial occupation had higher odds of utilising SRH referral services compared to women with partners who had no occupation (aOR = 3.11, 95% CI 1.14–8.45).

### Utilisation of IPV services

Table 6 shows the factors associated with utilisation of IPV services among currently partnered women in high migration communities of six Southern African countries. Migration status was significantly associated with utilization of IPV services (*P* = 0.045). Compared to non-migrants, both internal and international women had lower odds of utilising IPV services (aOR = 0.53; 95% CI 0.18–0.95) and (aOR = 0.34; 95% CI 0.10–1.12) respectively. Age was significantly associated with utilisation of IPV services (*P* = 0.025). After controlling for other factors, a five-year increase in age increased the odds of utilising IPV services by 34% (aOR = 1.34, 95% CI 1.04–1.74).

**Table 6:** Factors associated with current utilisation of IPV services among partnered women in high migration communities of six Southern African countries

Variable	Level	Prevalence of utilisation of IPV services	Unadjusted OR(95% CI)	Adjusted OR (95% CI)	*P*-value
		***n* = 204(39)**		***N* = 340**	
**Migration status**	Non-migrant	144(39.9)	1.00	1.00	0.045
	Internal migrants	39(39.0)	0.96(0.61–1.52)	0.53(0.18–0.95)	
	International migrant	21(32.8)	0.74(0.42–1.29)	0.34(0.10–1.12)	
**Duration of stay in community**			1.06(0.99–1.14)	0.90(0.73–1.11)	0.32
**Country**	South Africa	18(39.1)	1.00	1.00	0.30
	Lesotho	11(45.8)	0.94(0.40–2.21)	1.72(0.39–7.51)	
	Malawi	76(44.2)	0.40(0.15–1.09)	2.28(0.73–7.13)	
	Mozambique	15(25.4)	0.76(0.28–2.06)	1.53(0.40–5.82)	
	Eswatini	28(27.7)	0.45(0.18–1.13)	1.53(0.45–5.27)	
	Zambia	56(45.5)	0.99(0.41–2.38)	3.32(1.07–10.30)	
**Age**			1.24(1.11–1.39)	1.34(1.04–1.74)	0.025
**Marital status**	Never married	60(27.5)	1.00	1.00	0.84
	Married	87(43.3)	2.01(1.34–3.02)	0.81(0.41–1.60)	
	Formerly married	57(53.8)	3.06(1.89–4.97)	0.83(0.38–1.84)	
**Educational level**	No education	16(33.3)	1.00	1.00	0.76
	Primary	80(42.1)	1.45(0.75–2.83)	0.96(0.33–2.78)	
	Secondary/higher	108(37.6)	1.21(0.63–2.30)	1.19(0.41–3.51)	
**Employment status**	Unemployed	80(39.2)	1.00	1.00	0.67
	Employed	124(60.8)	1.78(1.24–2.52)	1.12(0.67–1.87)	
**Religion**	Catholic	52(34.4)	1.00	1.00	0.0
	Other Christians	135(41.0)	1.32(0.89–1.98)	1.52(0.85–2.72)	
	Other religion	17(37.8)	1.16(0.58–2.30)	1.46(0.51–4.24)	
**Comprehensive knowledge about SRH**			1.06(0.72–1.56)	0.82(0.44–1.52)	0.89
**Comprehensive knowledge about HIV**			1.43(1.14–1.79)	1.41(0.99–2.01)	0.13
**Parental violence**	No	128(39.0)	1.00	1.00	0.46
	Yes	76(38.0)	0.96(0.66–1.37)	0.83(0.50–1.37)	
**Attitude towards beating**	No	119(48.0)	1.00	1.00	0.001
	Yes	85(31.0)	0.49(0.34–0.69)	0.39(0.22–0.70)	
**Partner’s age**	< = 24	41(36.6)	1.00	1.00	0.9
	24–34	62(37.8)	1.05(0.64–1.73)	1.18(0.60–2.32)	
	> = 35	54(49.1)	1.67(0.98–2.85)	1.40(0.58–3.35)	
	Don’t know	39(41.9)	1.25(0.71–2.20)	1.20(0.52–2.78)	
**Partner’s educational level**	No education	39(32.0)	1.00	1.00	0.69
	Primary	51(50.5)	2.17(1.26–3.74)	1.44(0.60–3.44)	
	Secondary/higher	108(41.1)	1.48(0.94–2.33)	1.29(0.61–2.73)	
**Partner’s occupation**	None	11(36.7)	1.00	1.00	0.20
	Agriculture	20(33.9)	0.89(0.35–2.22)	0.74(0.22–2.51)	
	Technical/managerial	17(27.9)	0.67(0.26–1.69)	0.36(0.10–1.25)	
	Skilled manual	47(42.0)	1.25(0.54–2.87)	0.90(0.31–2.65)	
	Unskilled manual	98(45.6)	1.45(0.66–3.19)	1.03(0.36–2.90)	
**SRH decision making power**			1.25(1.12–1.40)	1.13(0.95–1.34)	0.15

Religion was marginally associated with utilisation of IPV services (*P* = 0.05). Compared to Catholic women, other Christians (aOR = 1.52; 95% CI 0.85–2.72) and Other religion (Traditionalists and Muslims) (aOR = 1.46; 95% CI 0.51–4.24) had higher odds of utilising IPV services. In addition, attitude towards wife beating was significantly associated with utilisation of IPV services (*P* = 0.001). Women who justified beating of a woman by her husband had 61% lower odds of utilising IPV services compared to women who condemn husbands beating their wives (aOR = 0.39, 95% CI 0.22–0.70). After adjusting for other factors, partner’s age, partner’s educational level, partner’s occupation and SRH decision making power were not significantly associated with utilisation of IPV services.

## Discussion

The study investigated the factors that are associated with utilisation of SRH services. The SRH services analysed are utilisation of modern contraceptive methods, utilisation of SRH referral services and utilisation of IPV services.

### Utilisation of modern contraceptive methods

Adjusting for other factors, there was no evidence of an association between migration status and non-utilisation of modern contraceptive methods. Country was found to be a significant factor with noticeable differences in utilisation of modern contraceptive methods among women indicating the different levels and strategies in the provision of modern contraceptive methods. Regarding marital status, the results of this study shows higher contraceptive method use among unmarried women which differs from the findings of a cross sectional study that was conducted among Chinese migrants where unmarried women were found to have lower odds of utilising modern contraceptive methods [38]. Similar to previous findings, women who used modern contraceptive methods were slightly younger on average than women who did not use modern contraceptive methods [14, 39]. In support of previous studies, employment status, comprehensive knowledge about SRH and comprehensive knowledge about HIV were found to be important factors that determine utilisation of modern contraceptive methods [38, 39]. This could be attributed to economic freedom, stability and empowerment that comes with having employment and SRH knowledge required to make appropriate decisions concerning their reproductive health and well as enforcing their reproductive health rights [39]. This reinforces the importance of disseminating accurate information about sexual and reproductive health to women. Regarding partner characteristic variables, partner’s age and partner’s educational level were found to be significantly associated with non-utilisation of modern contraceptive methods. This strengthens the importance of partner’s support and understanding of the importance of contraception and household economic stability in utilisation of SRH services.

### Utilisation of SRH referrals

After controlling for other factors, there was no evidence of an association between migration status and utilisation of SRH services. The significant association between ever denied access to a public healthcare facility and utilisation of SRH referral services shows unexpected results with women who were once denied access to a healthcare facility having higher odds of utilising SRH referral services. Utilisation of SRH referral services varied by country thereby revealing the differentials in cultural, legal, socio-economic development and service provision across the countries. In addition, the study found that married women and formerly married women had higher odds of utilising SRH referral services.

### Utilization of IPV services

The lower odds of utilising IPV services among migrant women is in line with several studies that found migration status to be an inhibiting factor in utilisation of IPV services. While this study did not explore the reasons for non-utilisation of IPV services, other studies have shown that language barriers, perceived inaccessibility of institutions, economic dependency on the partner, fear of deportation among international migrants and perceived discrimination by service providers [40–45]. Similarly, the results are in line with the findings of a study on determinants and barriers of patient-clinician communication about IPV where migration status was found to be a factor that hinder women from utilising IPV services in clinics [43, 46].

The significance and inhibiting influence of witnessing family violence and residing in communities that accept wife beating reflects the shared and prevailing cultural norms in many African countries that normalise the experience of violence from an intimate partner [42, 47–54]. As a result, utilization of IPV services from the police and other law enforcement agencies is viewed as disrespectful by families and communities [55]. The significance of age reflects the changes in women’s autonomy as they grow older, become educated and attain economic stability thereby empowering them to make independent decisions regarding their SRH issues. The significance and inhibiting influence of religion to utilisation of IPV services among Catholic women support the findings of a study conducted in Nigeria which showed that Catholic women were less likely to utilise IPV services [55]. This was mainly attributed to common religious teachings of female submission and obedience to their partner as the head of the family [49–52, 55–57].

### Strengths and limitations of the study

This study is drawn from a multi-country survey conducted in six Southern African countries, one of the few surveys that collected data on sexual and reproductive health of women in high migrant communities in Southern Africa. It explored a range of major reproductive health service components as they relate to migration indicators, a field of inquiry previously underexplored in Southern Africa. However, the study is not exempt from limitations, secondary data analysis does not provide the researcher with control over the data items that were collected, which are the sole use of existing data rather than complementing it with primary data in which questions that are more closely related to research questions could have been asked of participants. Intimate partner violence is a sensitive issue and social stigma is attached to it, hence respondents may not report their actual experience and help-seeking of IPV, thus the study is subject to social desirability bias. In addition, women were asked about their partner’s age and partner’s educational level and may not remember the correct age and educational level of their partners. Similarly, younger respondents with older partners and older respondents with younger partners may intentionally provide incorrect age of their partners in order to avoid embarrassment. This survey is cross-sectional in nature, hence the causal relationship between explanatory variables and outcome variables cannot be established.

## Conclusion

Migration status impacts on the way women utilise sexual and reproductive health services. Sexual and reproductive health awareness campaigns that target all women regardless of migration status are crucial in educating migrants about the available IPV services and their rights to equitable access to SRH services. The findings of the study can be used by SRH program managers to shape the development of SRH service provision and promotion programs. For example, community SRH promotion can address community perceptions about wife beating and religious beliefs to increase approval of modern contraceptive use, utilisation of SRH referral services and utilisation of IPV services. It is important to educate religious leaders so that they can participate in community awareness mainly around issues of IPV so that they can educate women about IPV within their churches using the faith based perspective. In addition, measures should be in place that ensure easy access to healthcare facilities and dissemination of information about side effects, actions to take and available options (informed consent) of contraception to promote utilisation of SRH services by all women regardless of their age. Devising and strengthening country specific strategies which are targeted at increasing utilisation of SRH services by women regardless of their migration status is crucial.

Training workshops, for example gender equality training that empower women psychologically, socially, economically and boost their self-esteem to decide about healthy SRH behaviours are required. This can be coupled with community mobilization and behaviour change strategies aimed at changing patriarchal social norms that justify wife beating. Initiatives that involve males and educate them about the importance of SRH services and the consequences of IPV are crucial. The findings highlight the need for strategies that tackle religious and cultural beliefs, age specific interventions, and country variations, encourages male involvement, women empowerment and autonomy in utilising SRH services regardless of their migration status.

## Supplementary Information

**Additional file 1:** **Supplementary** **Table 1.** Factors associated with current non-use of modern contraceptive methods among partnered women in high migration communities of six Southern African countries. **Supplementary Table 2.** Factors associated with utilisation of SRH referral services among partnered women in high migration communities of six Southern African countries.

## Data Availability

The SRHR-HIV Knows no Borders Project data is not publically available. It is available from the SRHR-HIV Knows no Borders Project consortium members (International Organization for Migration (IOM), Save the Children Netherlands (SCN) and University of the Witwatersrand’s School of Public Health (WSPH)) upon request by emailing Prof. Latifat Ibisomi Latifat.Ibisomi@wits.ac.za and Dr Francis Mulekya fbmulekya@iom.int or ropretoria@iom.int.

## Abbreviations

HIV: Human immunodeficiency virus
IOM: International Organization for Migration
IPV: Intimate partner violence
SCN: Save the Children Netherlands
SRH: Sexual and reproductive health
SRHR-HIV: Human immunodeficiency virus; Sexual and reproductive health and rights
WSPH: University of the Witwatersrand’s School of Public Health
