# Supplementary material for: Factors associated with sexual and reproductive health service utilisation in high migration communities in six Southern African countries
Source: BMC Public Health. 2022 May 2;22:876. doi: 10.1186/s12889-022-13308-4 (PMC9063360; doi:10.1186/s12889-022-13308-4)
Supplement: Supplementary file 1 — Additional file 1: Supplementary Table 1. Factors associated with current non-use of modern contraceptive methods among partnered women in high migration communities of six Southern African countries. Supplementary Table 2. Factors associated with utilisation of SRH referral services among partnered women in high migration communities of six Southern African countries. [file 12889_2022_13308_MOESM1_ESM.docx]

# **Supplementary tables**

**Table 1: Factors associated with current non-use of modern contraceptive methods among partnered women in high migration communities of six Southern African countries.**

|  |  | **Prevalence of modern contraceptive non-use n (%)** | **Unadjusted OR (95% CI)** | **AOR (95% CI)** | **P-value** |
| --- | --- | --- | --- | --- | --- |
| **Variable** | **Level** | **566(37.8)** |  | **N=952** |  |
| **Migration status** | Non-migrant | 388(38.8) | 1.00 | 1.00 | 0.815 |
|  | Internal migrants | 101(33.3) | 0.79(0.60-1.03) | 0.84(0.44-1.58) |  |
|  | International migrants | 77(39.5) | 1.03(0.75-1.41) | 0.97(0.49-1.93) |  |
| **Duration of stay in community** |  |  | 1.05(1.01-1.09) | 1.04(0.92-1.17) | 0.514 |
| **Country** | South Africa | 76(35.4) | 1.00 | 1.00 | 0.002 |
|  | Lesotho | 90(45.2) | 1.51(1.01-2.24) | 1.92(1.04-3.56) |  |
|  | Malawi | 113(30.4) | 0.80(0.56-1.14) | 0.84(0.47-1.52) |  |
|  | Mozambique | 119(42.2) | 1.34(0.93-1.93) | 0.66(0.36-1.21) |  |
|  | Eswatini | 62(40.0) | 1.22(0.80-1.87) | 1.50(0.78-2.88) |  |
|  | Zambia | 106(38.6) | 1.15(0.79-1.66 ) | 0.83(0.45-1.56) |  |
| **Age** |  |  | 1.10(1.04-1.17) | 1.19(1.02-1.39) | 0.027 |
| **Marital status** | Never married | 201(35.8) | 1.00 | 1.00 | 0.152 |
|  | Married | 245(37.3) | 1.07(0.84-1.35) | 1.31(0.88-1.94) |  |
|  | Formerly married | 120(42.9) | 1.34(1.00-1.80) | 1.63(0.99-2.69) |  |
| **Educational level** | Primary/lower | 257(40.9) | 1.00 | 1.00 | 0.229 |
|  | Secondary/higher | 306(35.4) | 0.79(0.64-0.98) | 0.8(0.58-1.14) |  |
| **Employment status** | Employed | 285(35.2) | 1.00 | 1.00 | P<0.001 |
|  | unemployed | 280(40.8) | 1.27(1.03-1.56) | 165(1.22-2.24) |  |
| **Religion** | Catholic | 197(40.5) | 1.00 | 1.00 | 0.417 |
|  | Other Christians | 312(35.9) | 0.82(0.66-1.03) | 0.97(0.70-1.34) |  |
|  | Other religion | 57(40.4) | 1.00(0.68-1.46) | 1.40(0.78-2.51) |  |
| **Desire for another child** | Have a child <2 years | 23(31.5) | 1.00 | 1.00 | P<0.001 |
|  | Have a child >=2 years | 80(31.1) | 0.98(0.56-1.72) | 1.08(0.53-2.19) |  |
|  | No more | 209(35.2) | 1.18(0.70-1.99) | 0.96(0.49-1.86) |  |
|  | Unsure | 14(37.8) | 1.32(0.58-3.03) | 1.70(0.59-4.91) |  |
|  | Missing | 240(44.7) | 1.76(1.04-2.96) | 2.38(1.22-4.68) |  |
| **Comprehensive knowledge about SRH** |  |  | 0.57(0.45-0.72) | 0.54(0.38-0.76) | P<0.001 |
| **Comprehensive knowledge about HIV** |  |  | 0.76(0.68-0.86) | 0.90(0.75-1.09) | 0.295 |
| **Partner’s age** | <=24 years | 144(40.1) | 1.00 | 1.00 | 0.013 |
|  | 25-34 | 168(34.6) | 0.79(0.60-1.05) | 0.74(0.49-1.10) |  |
|  | >=35 | 120(35.2) | 0.81(0.60-1.10) | 0.54(0.33-0.90) |  |
|  | Don’t know | 134(43.0) | 1.12(0.83-1.53) | 0.66(0.40-1.10) |  |
| **Partner’s educational level** | Primary/lower | 247(42.3) | 1.00 | 1.00 | 0.050 |
|  | Secondary/higher | 311(34.6) | 0.72(0.58-0.90) | 0.72(0.50-0.99) |  |
| **Partner’s occupation** | Agriculture | 68(39.8) | 1.00 | 1.00 | 0.736 |
|  | None | 43(43.4) | 1.16(0.70-1.92) | 1.19(0.61-2.31) |  |
|  | Technical/managerial | 82(34.3) | 0.79(0.53-1.19) | 0.99(0.55-178) |  |
|  | Skilled manual | 120(36.1) | 0.86(0.59-1.25) | 1.23(0.72-2.09) |  |
|  | Unskilled manual | 200(36.4) | 0.87(0.61-1.23) | 0.95(0.59-1.52) |  |
| **Experience of IPV** | Yes | 140(31.6) | 1.00 | 1.00 | 0.429 |
|  | No | 366(39.0) | 1.38(1.09-1.76) | 1.14(0.82-1.62) |  |
| **SRH decision making power** |  |  | 0.96(0.89-1.04) | 0.97(0.87-1.09) | 0.665 |

**Table 2: Factors associated with utilisation of SRH referral services among partnered women in high migration communities of six Southern African countries.**

|  |  | **Prevalence of utilisation of SRH referral services** | **Unadjusted OR (95% CI)** | **AOR (95% CI)** | **P-value** |
| --- | --- | --- | --- | --- | --- |
| **Variable** | **Level** | **N=319(48.3)** |  | **N= 425** |  |
| **Migration status** | Non-migrant | 200(45.2) | 1.00 | 1.00 | 0.544 |
|  | Internal migrants | 49(55.1) | 1.49(0.94-2.35) | 1.29(0.49-3.42) |  |
|  | International migrants | 70(54.7) | 1.47(0.99-2.18) | 0.80(0.30-2.18) |  |
| **Duration of stay** |  |  | 0.96(0.91-1.02) | 0.88(0.73-1.07) | 0.223 |
| **Ever denied access to a public healthcare facility** | No | 196(42.6) | 1.00 | 1.00 | P<0.001 |
|  | Yes | 123(61.5) | 2.15(1.53-3.02) | 3.15(1.69-5.77) |  |
| **Country** | South Africa | 61(64.9) | 1.00 | 1.00 | P<0.001 |
|  | Lesotho | 0.0(0.0) | empty | Empty |  |
|  | Malawi | 78(45.4) | 0.45(0.26-0.75) | 0.22(0.09-0.56) |  |
|  | Mozambique | 50(35.5) | 0.30(0.17-0.51) | 0.36(0.14-0.92) |  |
|  | Eswatini | 78(52.7) | 0.60(0.35-1.03) | 0.45(0.19-1.05) |  |
|  | Zambia | 52(53.1) | 0.61(0.34-1.09) | 1.58(0.58-4.78) |  |
| **Age** |  |  | 1.09(0.99-1.20) | 1.19(0.94-1.51) | 0.142 |
| **Marital status** | Never married | 127(45.4) | 1.00 | 1.00 | 0.049 |
|  | Married | 133(49.4) | 1.18(0.84-1.65) | 1.26(0.71-2.23) |  |
|  | Formerly married | 59(53.2) | 1.37(0.88-2.12) | 2.68(1.22-5.90) |  |
| **Educational level** | Primary/lower | 125(43.3) | 1.00 | 1.00 | 0.294 |
|  | Secondary/higher | 193(52.2) | 1.43(1.05-1.95) | 1.32(0.79-2.20) |  |
| **Employment status** | Unemployed | 171(50.2) | 1.00 | 1.00 | 0.064 |
|  | Employed | 148(46.5) | 0.87(0.64-1.18) | 0.65 (0.41-1.03) |  |
| **Religion** | Catholics | 89(46.4) | 1.00 | 1.00 | 0.306 |
|  | Other Christians | 194(48.7) | 1.10(0.78-1.55) | 0.82(0.49-1.36) |  |
|  | Other religion | 36(51.4) | 1.23(0.71-2.12) | 1.42(0.62-3.24) |  |
| **Comprehensive knowledge about SRH** |  |  | 1.30(0.94-1.80) | 1.15(0.69-1.91) | 0.907 |
| **Comprehensive knowledge about HIV** |  |  | 1.22(1.02-1.45) | 1.25(0.92-1.68) | 0.193 |
| **Partner’s Age** | <=24 | 75(46.0) | 1.00 | 1.00 | 0.759 |
|  | 25-34 | 101(52.6) | 1.30(0.86-1.98) | 1.17(0.64-2.11) |  |
|  | >=35 | 83(57.2) | 1.57(1.00-2.47) | 1.04(0.49-2.19) |  |
|  | Don’t know | 59(37.1) | 0.69(0.44-1.08) | 0.80(0.38-1.68) |  |
| **Partner’s educational level** | Primary/lower | 134(45.3) | 1.00 | 1.00 | 0.841 |
|  | Secondary/higher | 183(50.8) | 1.25(0.92-1.70) | 1.05(0.62-1.78) | 0.841 |
| **Partner’s occupation** | None | 19(40.4) | 1.00 | 1.00 | 0.101 |
|  | Agriculture | 38(45.2) | 1.22(0.59-2.51) | 1.46(0.53-4.04) |  |
|  | Technical/managerial | 63(63.0) | 2.51(1.23-5.10) | 3.11(1.14-8.45) |  |
|  | Skilled manual | 64(46.4) | 1.27(0.65-2.50) | 1.31(0.52-3.28) |  |
|  | Unskilled manual | 113(49.3) | 1.44(0.76-2.72) | 1.91(0.81-4.50) |  |
| **SRH decision making power** |  |  | 0.90(0.81-0.99) | 0.95(0.81-1.12) | 0.540 |
| **Experience of IPV** | No | 145(44.5) | 1.00 | 1.00 | 0.269 |
|  | Yes | 152(56.9) | 1.65(1.19-2.29) | 1.35(0.81-2.27) |  |
